# Reclaiming the concept of professionalism in the digital context: a principle-based concept analysis

**DOI:** 10.1080/07853890.2024.2398202

**Published:** 2024-09-12

**Authors:** Shaista Salman Guraya, Salman Yousuf Guraya, Fiza Rashid-Doubell, Salim Fredericks, Denis W. Harkin, Mohd Zarawi bin Mat Nor, Muhamad Saiful Bahri Yusoff

**Affiliations:** a Institute of Learning Mohammad Bin Rashid, University of Medicine and Health Sciences, Dubai Healthcare City, Dubai, Unted Arab Emirates; b College of Medicine, University of Sharjah, Sharjah, Unted Arab Emirates; c Professor of Physiology (Retired), Edinburgh, UK; d Royal College of Surgeons Ireland, Bahrain RCSI–MUB, Adliya, Bahrain; e Faculty of Medicine Health Sciences, Royal College of Surgeons in Ireland, Dublin, Ireland; f Department of Medical Education, School of Medical Sciences, Universiti Sains Malaysia, Kubang Kerian, Malaysia

**Keywords:** Concept analysis, principle-based concept analysis, e-professionalism, professionalism, digital context

## Abstract

**Background:**

There has been an alarming surge in the usage of social networking sites (SNSs) by healthcare professionals (HCPs) without adherence to the principles of professionalism. The widespread use of SNSs in medical practices has been coupled with reports of breaches of professional behaviors. Despite the benefits of SNSs, skepticism prevails about a clearly defined role for SNSs within medicine based upon the core principles of professionalism. Thus, there is a need to understand the manifestations of professionalism in the digital context, classically known as e-professionalism. This study systematically examines HCPs’ perceptions of e-professionalism to advance a thorough understanding of e-professionalism.

**Methods:**

This concept analysis was performed using the principle-based approach of Penrod and Hupcey. In January 2023, we searched the databases of PubMed and ISI Web of Science for English-language articles specific to ‘e-professionalism’ in the medical field. The final selected research corpus of 63 articles was analyzed in this study.

**Results:**

A comprehensive analysis of the selected articles highlighted that e-professionalism is an epistemologically mature and distinct concept by a standard definition. However, inconsistencies in conceptual meanings were reported due to varied interpretations despite digital literacy. The pragmatic utility showed a lack of sound methodological and philosophical paradigms. Perhaps the rapid technological advancements and manifestations have hampered linguistic maturity. However, logically, e-professionalism is perceived as an extension of conventional professionalism but with a focus on a distinct framework with a set of attributes to be digitally relevant.

**Conclusion:**

This study identifies a scarcity of research about the collective perspective of essential stakeholders, underpinning the need to further explore e-professionalism due to its emerging complex nature within the digital context. There is also a recognition that a framework is essential to guide future HCPs to yield a profound understanding and to provide remediation strategies in the rapidly advancing medical field in digital realm.

## KEY MESSAGES

- This principle-based concept analysis provides a nuanced understanding of e-professionalism, addressing its theoretical integration, practical utility, linguistic challenges, and logical boundaries.
- E-professionalism is acknowledged as a distinct concept across medical disciplines but variations in its interpretation demands a standard, unified, and multi-construct framework.
- A wealth of generational perceptions surround the concept of e-professionalism, including digital literacy and competency gap, signifying the importance of tailored educational strategies to ensure healthcare professionals uphold high standards while practicing in the digital realm.

## Introduction

Alongside the enormous proliferations of social media networking worldwide, we are witnessing the rising popularity of Social Networking Sites (SNSs) among Health Care Professionals (HCPs) [1]. The exponential growth and evolution of SNSs platforms (e.g. Facebook, Twitter, LinkedIn, Instagram, etc.) have grown a large community of end-users where usage takes place without adherence to core principles of medical professionalism (REF). SNSs are more cost-effective than conventional ­communication tools (e.g. mobile technology), and provide direct access to masses irrespective of geographical boundaries with communication taking place in *‘real-time’* [1,2]. Though there are several obvious benefits of SNSs for the medical fraternity including physicians, medical faculty, and students, including networking, outreach, connectivity, targeting large audiences instantly, medical educators remain sceptical about their regulated and controlled applications [2].

In the medical field, SNSs are commonly used for sharing information, medical education, and for a reciprocal communication between doctors, patients and for campaigns to promote health and prevent disease. A rise in the usage of SNSs for such activities has led to a multitude of publications reporting breaches of professional values and behaviours in the digital realm [3,4]. These reports alert medical educators and policy makers to consider the unique specifications and regulations to safeguard professional behaviour online or e-professionalism; the manifestation of standard professionalism in SNSs. More importantly, *‘given the capability of social media to reach a large audience, gaffes can be quickly communicated and image, reputation, and professional standing damaged’* [5]. Unfortunately, e-professionalism does not provide a clear understanding of conduct while online and can lead to discordance within the profession. Thus underpinning the need to develop the conceptual basis of e-professionalism in medical education.

Despite the growing interest in the concept of ‘e-professionalism’ in medical education, exploratory research pertaining to its explicit definitions, boundaries, measurements, and fundamental attributes remains scarce. In spite of a substantial body of work on e-professionalism, there remains an enormous knowledge gap within medical education about this emerging subject. In addition, there is a necessity to introduce digital awareness of appropriate behaviours and communication for both digitally naïve and native medical professionals as well as students. Furthermore, a clear, precise, and explicit understanding of the current knowledge, with theoretically and scientifically sound research is essential. Hence, the adoption of a concept analysis approach was deemed suitable to achieve our objectives. This concept analysis aims to examine the perceptions and understandings of current and future HCPs about regarding e-professionalism in a systematic and robust manner. Additionally, we aim to explore the nuanced aspects of safeguarding medical professionalism in the digital context, contributing to a more accurate depiction of this concept within the landscape of medical education.

## Materials and methods

### Framework for concept analysis

The study uses a concept analysis approach. A concept is a *‘word or phrase summarizing a phenomenon, an idea, an observation, or an experience’* [6]. Furthermore, the validation of concepts in the medical field is critical; e-professionalism demonstrates a phenomenon observed in educational and clinical practice. This process, in turn, has implications for the pattern of practice of HCPs. Finally, to guide the scientific development and validation of the concept, it should represent a real phenomenon. There should also be evidence that the concept is relevant to clinical practice [7]. A wide range of models are available in the literature describing how concept analysis can be conducted; the evolution approach [8], the Walker and Avant [9] eight-step process, and the Penrod and Hupcey [10] principle-based method of concept analysis. The latter method of Penrod and Hupcey [10] differs from others because it directs us towards the most suitable strategies for concept advancement and research design.

**Figure 1.**
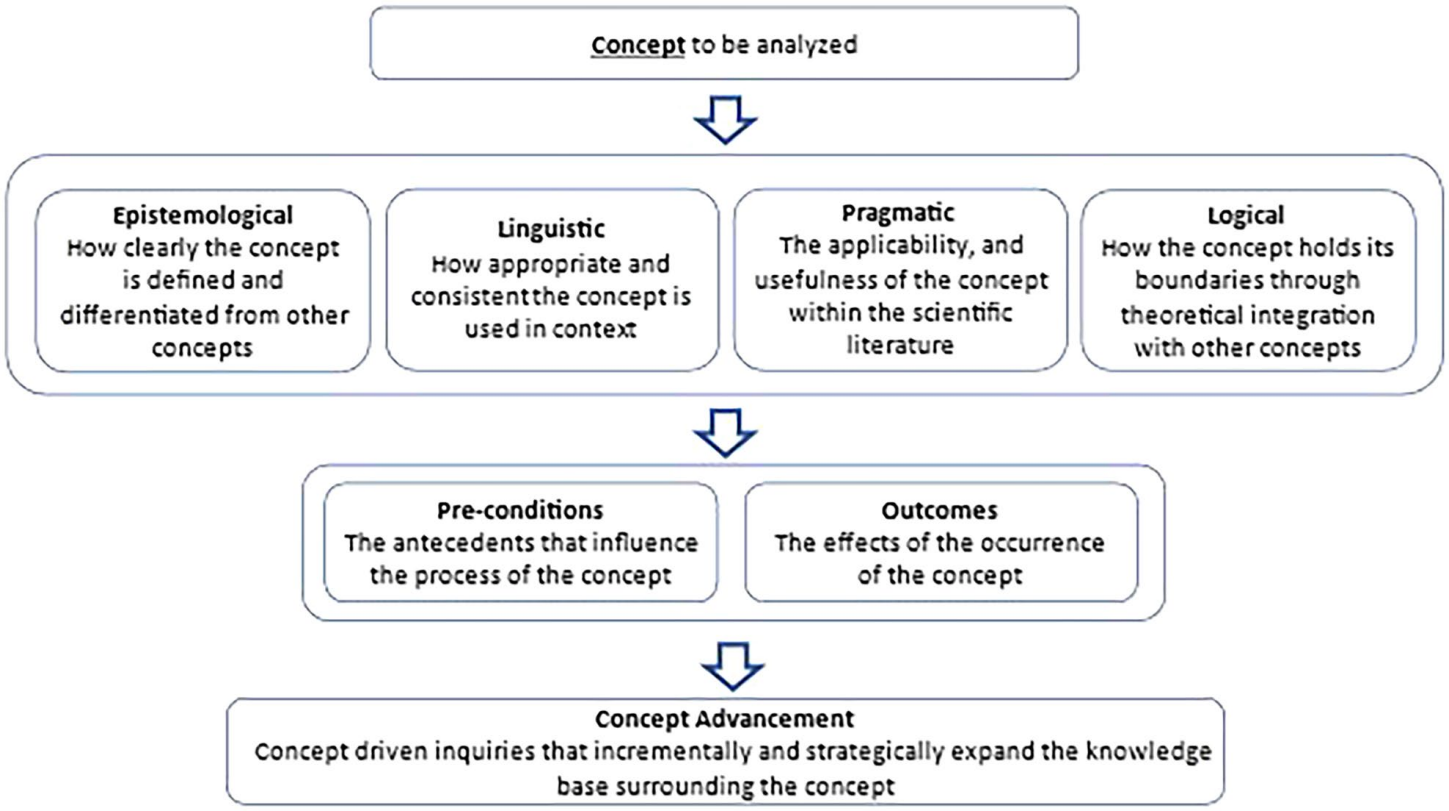
The principle-based concept analysis guiding framework of penrod and hupcey [10].

The principle-based concept analysis method helps researchers to collate the existing body of literature based on epistemological, pragmatic, linguistic, and logical principles of philosophy. It is a deductive strategy, but inductive insights may arise during the analysis, allowing nuances and complexities to be revealed in the data analysis phase [11]. The strength of this approach is that it helps the researcher to synthesize and create a theoretical model to advance current knowledge in the selected subject. The principle-based concept analysis provides a structured and well-organized framework to conduct concept analysis [12,13]. We used the principle-based method of concept analysis to synthesize and summarize the findings of the existing literature on e-professionalism [10,14] (Figure 1).

The overarching purpose of the concept analysis was to produce evidence to best fit the *‘evidence-based truth’* in the scientific literature. Each principle was scrutinised further using Morse’s level of maturity, ranging from under-developed to fully developed [15]. The evaluation criteria included an assessment of concept definition, characteristics of the concept, and conceptual boundaries. Since the term professionalism is clearly understood in the literature, we focussed our attention on advancement e-professionalism.

### Sampling and selection of literature

We included published work about *‘e-professionalism’* without any time constrains. The criteria for the selection of articles included the English-language articles published in peer-reviewed journals about ‘e-professionalism’. We searched the current sample based on nature of the digital context and SNSs and professional roles in the digital context to evaluate the current knowledge about e-professionalism and its related constructs. Abstracts, personal statements, or the articles without full text were excluded.

### Sampling technique

**Figure 2.**
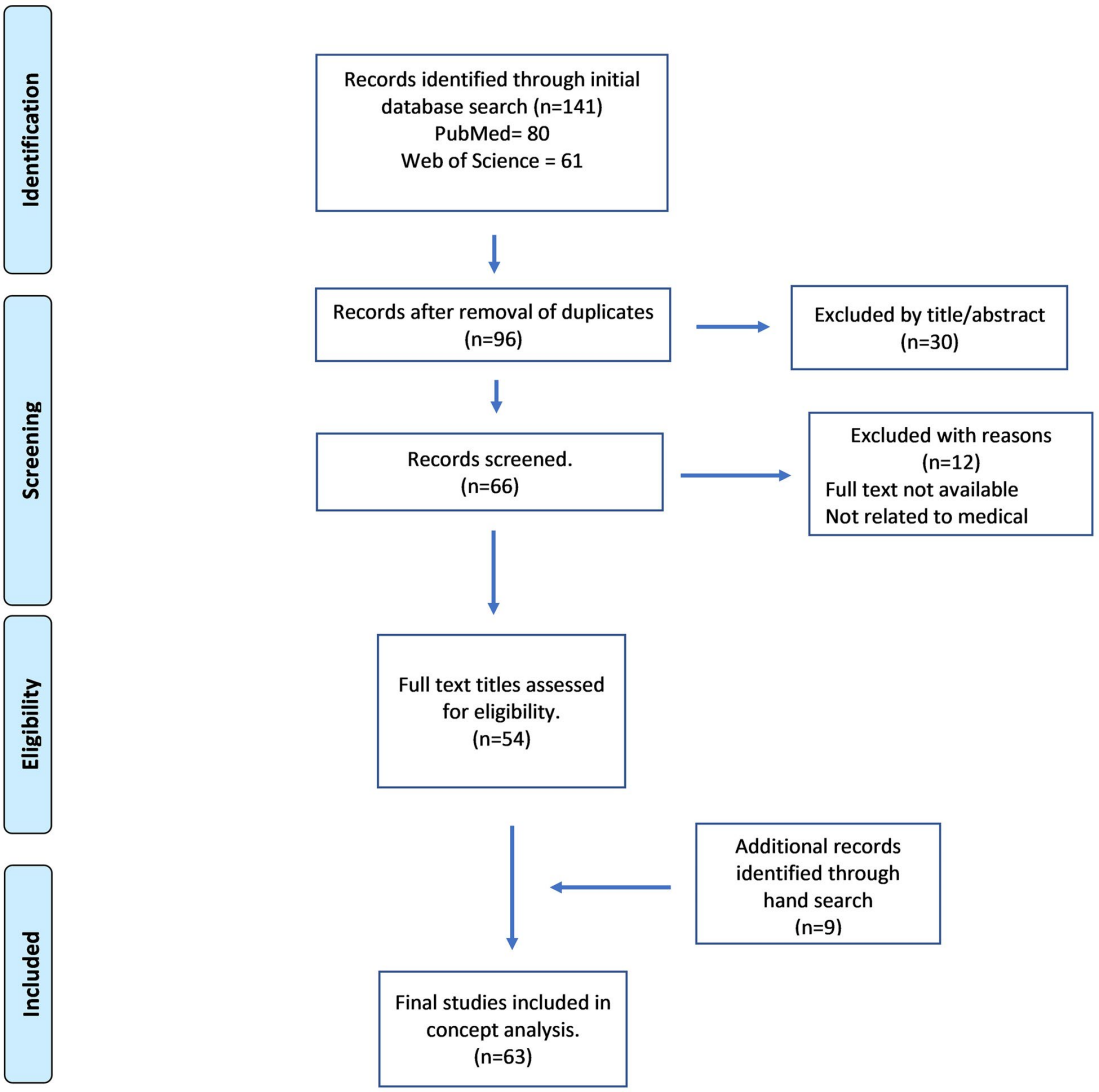
An illustration of the systematic literature search with results.

In January 2023, using a purposive sampling technique complemented by citation tracking, a search of PubMed and Web of Science using the key term ‘e-professionalism’ according to the inclusion criteria illustrated in (Figure 2) was undertaken.

An initial yield of 141 articles were retrieved based on the inclusion criteria. After removing the duplicates, initial screening was performed by reviewing the title and abstracts. A second screening of articles not specifically relevant to the concept of e-professionalism were eliminated. Nine references were added after the hand search and the final data set included 63 references in total [2,4,5,16–75].

### Data extraction and analysis

Using the four principles of Penrod and Hupcey [10], a priori table was developed for data extraction (Table 1).

**Table 1.** Prompts used in the data extraction to ascertain the principles of concept analysis.

Principles	Questions
Epistemological	What is the definition and definitional elements of e-professionalism in medical education? Is e-professionalism well differentiated from other related concepts? What are the other related concepts mentioned?
Pragmatic	What is the use of e-professionalism in clinical/academic practice? What are the uses for research? How has e-professionalism been operationalized?
Linguistic	What is the linguistic consistency around e-professionalism? What is the linguistic maturation and appropriateness within the context?
Logic	Does the e-professionalism have distinct boundaries as a concept? What is the evidence about integration of e-professionalism from theoretical perspectives? Has the concept been appropriately operationalized? What are the key characteristics of e-professionalism? What is the evidence about the consistent use of those key characteristics?

All included articles were critically analyzed against four principles: explicit and implicit meaning of e-professionalism (epistemology), usage of e-professionalism (pragmatics), consistency in application and meaning (linguistic), and acculturation of e-professionalism concept from relevant literature (logic). We examined and documented all pertinent dimensions in the priori table, which were later ­assimilated into the results after repeated deliberations. All authors reviewed the completed priori table and interpretation of the results. After repeated deliberations keeping all four principles in mind, all authors critically revised the data entry and its relevance to the synthesis of this literature using a constant comparing and contrasting technique.

While focusing on the purpose of inquiry, the rationality of the searched topic highlighted significant analytical questions. During the synthesis of results, a critical examination of explained definitions, their implied conceptual meaning, application of e-professionalism as a phenomenon of interest were sought carefully as outlined in Table 1. A summative conclusion was yielded by a detailed data analysis while collating, comparing, and contrasting the key trends which dissected the maturity level of each principle.

### Methodological rigor

To eliminate research bias and to ensure that a representative sample of pertinent literature was collected, we used a reflexive approach by reading each article twice by the principal investigator. This method has been reported to minimize the possibility of research bias and to ensure that the article collection was representative of the selected discipline [76]. Also, the principal investigator had prior experience in the research process. This expertise shaped assumptions and presuppositions, potentially impacting on the data interpretation. Thus influencing the overall transferability of the study’s findings. As per the Standards for Reporting Qualitative Research (SRQR) [76] guidelines, we acknowledged the inherent challenges in creating and applying reporting criteria in qualitative research when compared to more structured frameworks found in quantitative studies (Supplementary Appendix 1). To avoid these caveats, we clearly delineated our framework-based methodology, and all the team members analyzed the four principles separately, thus enhancing the reliability and validity of our findings.

## Results

### Epistemological principle: Is the concept clearly defined and distinct from other concepts?

Epistemology, is a philosophical approach to inquiry pertaining to the nature and conditions of knowledge, essentially clarifying how well a concept is distinguished from others [77]. Cain [17] defined e-professionalism as *‘the attitudes and behaviours (which may occur in private settings) reflecting traditional professionalism paradigms manifested through digital media’*. Published literature appears to have reached a consensus on this definition. However, Cleary [26], by using the term *‘netiquette’,* extended its range beyond simple online communication.

#### Conceptual foundation of e-professionalism

In an attempt to understand the epistemological maturity, we focused on what constituted as unprofessional online behaviour. Despite a clear definition, we found diverging opinions between students and physicians [70]. Current literature shows an agreement that socialization to professional behaviours is an important feature, but itself is an elusive concept with ambiguous boundaries. Intricate conversations, interactions, and socialization in the open world of web has distinctly blurred the divide between public and private lives by sharing personal information [16,20,71]. Consequently, professionalism lapses in the electronic realm include inadvertent postings of photos; disclosing social and political affiliations; personal and religious preferences which are not generally revealed in a conventional doctor-patient relationship; disrespectful behaviours, and poor self-awareness [22,57,66,67]. Ellaway [34] argued that breaches in e-professionalism were analogous to a blurring of personal and professional boundaries. At the same time, others have shown that intersecting personal and professional identities on SNSs pose challenges to personal and patient privacy (REFS). Cain [16] highlighted the importance of SNSs in the life of an information-fluent generation and an understanding of underlying mechanisms of ‘web works’ often threatening the trust and privacy online by posing risk, liability, and copyright issues. However, it can be argued that the onus of privacy protection lies on the person posting the information by explicitly stating the expectations (REF). Benetoli [44] reported that unprofessional behaviours by HCPs on SNSs may influence public perceptions of their medical professionalism. Good patient care is built on strong professional values and behaviour and unprofessional actions online generally reflect the erosion of ethical boundaries [50]. Every day digital natives face new risks for behaving in an unprofessional and unethical manner while using electronic medical records and social media.

#### Challenges in defining unprofessional behaviours in the cyber space

Upon examination through the epistemological lens, literature portrays a varied range of perceptions revealing inadequate awareness of the caveats and risks of the digital world. This makes students in particular vulnerable and subjects them to an increased probability of engaging in unprofessional online activities ­compared to qualified HCPs [25]. Zalpuri [71] and Rukavina [2] described some important elements of e-professionalism ranging from confidentiality, boundaries issues, advocacy, netiquettes, integrity, and loosening accountability. More recently, Rukavina [74] devised a coding scheme to categorize unprofessional behaviours on SNSs with an intention to reduce the influence of subjective interpretation.

#### Navigating privacy and identity in SNSs

The literature has consistently suggested that SNSs constitute rapid, interactive, and modern ways of communicating, educating, and advocating for patients but have the potential to compromise their privacy and confidentiality [2,30]. Additionally, an added layer of complexity is recognized due to the generational differences in the use, perception, and acceptance of SNSs. An appreciation that current learners are adept at sharing liberal information manifests as the same attitudes and behaviours to all opportunities to share digital information, leading to blurring personal and professional lives. Kaczmarczyk [28] has proven that digital natives lack insight into their professional identities while using SNSs. This narrative was reinforced recently by the emerging importance of *‘context’*, which in our case is the digital world, thus, requiring a new set of regulations and guidelines for web-based interactions [74]. The absence of healthcare students regard for appropriate professional SNSs postings is noteworthy [4]. The impact on well-being associated with the excessive use of SNSs includes social disconnection, mental and physical stress, and societal disconnect (REF). A consistent use and teaching of the concept of e-professionalism to provide a regulated and conscientious use of technology to safeguard both personal and professional identities are essential [63].

#### Educational strategies and frameworks for navigating social media

Within the epistemological framework, another key challenge is to safeguard the societal and professional contract between doctor and patient. A lack of awareness by HCPs while using SNSs, such as Facebook makes them professionally vulnerable online [63]. Online platforms pose significant ethical risk that many users do not comprehend due to an absence of confidentiality proficiency. A well-structured strategy to teach e-professionalism within health professions education is an essential next-step [4,63]. Its need is evidenced by the inconsistent responses to digital misdemeanours at institutional level manifests as a binary approach without considering nuances or alternative approaches [78], creating a hidden curriculum of digital unprofessionalism. However, the reviewed literature shows the development of a novel framework aimed at the transpersonal level integrating the concept of *‘Mission’* into pre-existing values, behaviours, and identity-based frameworks. This framework enumerated the desired attributes, providing guidance to good e-professional behaviour [62].

### Pragmatic principle: Is the concept relevant and practical within the scientific realm of inquiry? Has it been in practice?

Pragmatism utilizes a discipline-specific concept evaluation to determine the pragmatic use and operationalization of the subject under consideration [14]. In essence, the pragmatic principle explores the efficacy of the concept under consideration and elaborates the phenomena in a discipline. This principle also demonstrates whether the published literature endorses and validates or limits a concept as useful and applicable and a useful entity in a field of interest [43]. We assessed the pragmatic utility of our concept by exploring the published literature for the concept evaluation and its operationalization in both academic and clinical fields.

#### Pragmatic assessment of e-professionalism

The need to evaluate the concept of e-professionalism dates back to Cain and Romanelli [17] and Aase [19] when patients feared that their private information was being published publicly, jeopardizing their confidence in HCPs. Patients became hesitant in sharing private medical information with their physicians [49]. The threat of online unprofessional contents being viewed by unknown audiences had legal and ethical ramifications including breach of patient confidentiality [24]. However, without a single validated and reliable tool of e-professionalism measurement, policing behaviour was difficult. In a study by Ness et al. [29], most students agreed that their online profile could influence public’s opinions about them and believed that as healthcare professional students they should navigate cyber space more cautiously. In several studies, self-administered questionnaires were used to gather information about digital literacy, use of SNSs, e-professionalism, and guidance on online communications and conduct [17,18,24,27,29,43,45]. Only two studies used qualitative methodology to determine a deeper understanding of the perceptions of pharmacists and nursing organizations, respectively [44,52].

#### Historical evolution and challenges

A deliberate exploration of selected studies in our analysis showed that the concept of e-professionalism focuses on three areas: the construction of e-professionalism instruments, its application in measuring professional behaviors, and its role within a medical curriculum (Table 2).

**Table 2.** The selected studies used in our review for pragmatic principle.

Pragmatic category		
Construction of e-professionalism instruments	Application of e-professionalism in measuring professional behaviours	e-professionalism concept—role within a medical curriculum
Study designs		
**Descriptive** Gettig, 2013 [27] Dobson, 2019 [54] Lee et al., 2019 [55] Lefebvre, 2020 [58] Karveleas et al., 2021 [65] Viskić et al., 2021 [70] Viskić et al., 2022 [75]	**Descriptive** Cain, 2009 [18] Cain, 2010 [20] Kjos, 2012 [24] Osman, 2012 [25] Ness et al., 2013 [29] Gupta, 2015 [35] Gettig et al., 2016 [39] Lefebvre et al., 2016 [41] Yang et al., 2016 [43] Duke et al., 2017 [45] Barnable, 2018 [49] Al-Qarni 2019 [53] Bacaksiz, 2020 [57] Griffin, 2021 [61] Hussain et al., 2021 [64] Nieminen et al., 2022 [73] O’Connor et al., 2022 [4] Kang et al., 2015 [36] **Qualitative** Bentoli et al., 2017 [44] Mather, 2018 [52] **Mixed-methods** Vukušić Rukavina et al., 2022 [74] **Reviews** Vukušić Rukavina et al., 2021 [2] Holden and Spallek, 2018 [50] Guraya, 2021 [63]	**Descriptive** Jackson, 2018 [51] Mostaghimi et al., 2017 [47] **Conference proceedings** John, 2012 [23] **Association Notes and Reviews** Spector et al., 2010 [22] Kaczmarczyk et al., 2013 [28] **Editorial and Commentary** O’Connor, 2020 [59] Arif, 2020 [56] Mattingly, 2010 [21] Cleary, 2013 [26] **Qualitative** O’Connor, 2020 [59] **Mixed-methods** Guraya et al., 2021 [62] Guraya et al., 2022 [72] **Case study** Orenstein, 2013 [30] Westrick, 2016 [42] Eijkholt, 2017 [46] Zalpuri et al., 2021 [71] Jones et al., 2016 [40] **Reviews** Cain, 2008 [16] Cain and Romanelli, 2009 [17] Aase, 2010 [19] Grindrod, 2014 [31] Benetoli, 2015 [33] Ellaway et al., 2015 [34] Kleppinger and Cain, 2015 [37] Neville and Waylen, 2015 [38] Schroeder, 2017 [48] Mosalanejad, 2021 [69]

Despite a consensus on the importance, value, and role of medical professionalism, e-professionalism has not been remitted to the same extent due to the emerging issues in the digital realm. Professional behaviour online is just beginning to be appreciated [28]. However, there is still an onus to empower digital natives users to make better choices while using SNSs to protect their own reputation and the interests of the public [2,31,37,38,48,69]. On a positive note, healthcare professions students have been willing to change how they used SNSs but need guidance and training. Overall pragmatism of being professional in the digital world was to practice personal and professional work by maintaining autonomy, and professional integrity while safeguarding societal contract, respect, and confidentiality.

#### Societal adjustment and academic leadership

Considering the pragmatic application of e-professionalism in societal spaces, our analysis proposes that academic leadership must define the values, standards, behaviours, policies, and best practices for SNSs usage [2,16,17,19,28,34,37,38,53,54,57,59,63,66,67,71]. Policies and statements describing e-professionalism were reported, however, due to the enormous proliferation of technological innovations it became evident that society needed time to adjust to the consequences of this new paradigm [30,46,50]. Philosophically, a large segment of our population struggles with how to work separately in public and private spaces while online [54]. Furthermore, there was a glaring lack of awareness regarding systems used to identify, track, and remediate professionalism lapses, made even harder by the differing generational perspectives defining appropriate professional behaviour [22,57–59]. Partly due to the difficulty in anticipating new technological advances. Spector et al. [22] reiterated the importance of e-professionalism in the era of technological advancements emphasizing the emerging challenges and threats to our professional standards and values. Some plausible recommendations included posting disclaimers, avoid diagnosing on SNSs, using the least biased educational material, and providing non-financial incentives in exchange of positive reviews or testimonials [30]. Even though e-professionalism is regarded as a pragmatically mature concept, a carefully drafted policy and use of checklists would be required for its application across social, cultural, economic, ethical, and political levels [22,42,56]. The paradigm shift of healthcare professionals from traditional healthcare settings to the broader social community is driven by their online presence, particularly on social networking sites (SNSs), which are recognized as valuable resources in all ­undergraduate medical and related programs [31,34,37,48,51,59,63].

#### E-professionalism in healthcare education

Focusing on the educational dimension, empirical research in the nursing field, both at system and organisation levels, identified a growing need for professional academic programs to develop specific guidelines for online behaviours and to educate students on how online behaviours were linked to the basic tenets of professionalism [2,4,39,42,45,49,51,57,58,61]. However, Gettig [27] focused on the importance of the faculty’s perspectives and understandings of digital media before educating students on the consequences of its misuse. Jones et al. [40] and Zalpuri [71] also suggested incorporating the use of Twitter, YouTube, and other SNSs into the medical curricula as way to upgrade the healthcare educators’ toolbox. In one example, Gettig et al. [51] used Twitter as an interactive and dynamic tool for education promotion and networking with nursing students, facilitating and promoting the digital professionalism learning experience. This kind of use and translation of the societal ethnography into cyberspace also permits the collection of data that is not free from bias and misbeliefs [50]. Therefore, a comprehensive investigation of the SNSs usage, the associated connotations on personal and professional dominions is required [45]. However, recent research produced a Medical Education e-Professionalism (MEeP) framework with theoretical underpinnings from the Theory of Planned Behaviour showed promising results in terms of behavioural changes in the participants [72].

### Linguistic principle: Is the concept used consistently and appropriately within context?

Linguistics is a robust and focused mechanism investigating the consistent use and meaning of the concept under consideration. The linguistic principle applies to the use of a subject to help evaluate its consistency [14,77,79]. The principle is also studied with regard to its context, whether it is wholly specific to a context or more abstract [14]. This principle corresponds to empirical testing to support the validity or invalidity of the designated assumptions or theoretical propositions [9].

#### Linguistic evaluation of e-professionalism

To examine the linguistic evolution of e-professionalism terminology, a search of published literature was conducted for the consistent use and meaning of e-professionalism. It was apparent that, linguistically, e-professionalism was a fledgling concept. Unfortunately, there was a lack of consensus about its nature and attributes needed to navigate the digital world respectfully. Although attributes of conventional professionalism in the online world seemed like an appropriate extension, the differences were remarkable when these attributes were considered within ‘private’ environments [17,62]. Two terms in e-professionalism that Duke et al. [45] highlighted ‘conduct unbecoming’ and ‘appropriate use of self-disclosure’ converged with conventional professionalism actions. Clearly indicating the non-binary nature of this multidimensional construct. Recent research reveals the significance of ‘context’ by applying a different set of rules and attributes while denoting the content and conduct unprofessional [62,63,74].

#### Evolving perspectives and grey areas

An individual’s online persona is considered a core element of e-professionalism and adds yet another dimension to several grey areas of e-professionalism [19,20]. These grey areas essentially stem from some contentious considerations; (1) whether evaluating alcohol or drug abuse from someone’s online persona could be considered as an e-professionalism issue and (2) how many online group affiliation violations should be used as a professionalism threshold? In essence, e-professionalism can be extrapolated as a modern extension of conventional professionalism grappling with issues of vagueness [17,24,42,47]. However, the development of a validated instrument to define potentially unprofessional behaviours with a sound focus on clear contextual understandings has been helpful [74]. Nevertheless, a rapid upsurge in new applications and platforms demands parallel innovative research to address foreseeable concerns and ambiguities that prevail around e-professionalism.

#### Cultural variances and educational interventions

A glaring finding in our review was an absence of comparison of cultural perspectives related to digital professionalism across medical education systems [56]. It was evident that policy makers were unable to influence guidelines on the professional use of SNSs. It was also evident that students were not aware of the proclaimed unethical behaviours on SNSs (REF). A large body of literature shows a conflict in the usage of SNSs due to cultural differences [25]. A standardization of the medical professionalism curricula to inculcate e-professionalism would be a way of negating these differences [35]. Gettig [27] suggested that ‘respect for others’ was an essential conventional professionalism attribute assisting students to develop as receptive professionals. Confounding variables for professionalism online necessitated a validated multi-construct Mission-based MEeP framework encompassing the desired attributes for digital professionalism [62,80].

To improve linguistic maturity, Kaczmarczyk’s [28] proposal to teach responsible use of SNSs through the development of an online professional persona seems reasonable. This strategy can enhance the professional behaviour of learners in this dynamic learning experience [37,38,72]. Curricular interventions introducing regulated professional use of SNSs provides learners with the right tools to navigate through potential pitfalls of the digital environment [51,56,71]. A constructive and comprehensive guidance for ‘digital professionalism’, is needed not just an adjunct to the existing concepts of professionalism [4,63,65]. Ellaway’s [34] review suggested insertion of the e-professionalism principles into the medical education setting through training and education of students and faculty of healthcare professions on appropriate self-disclosures. Others demand dedicated training on e-professionalism in medical curricula for faculty and students [2,47]. One challenge is Keeping the rapidly technological evolution in focus, their research coined the use of behaviourally oriented practices, such as ‘encrypting mobile devices’ as opposed to ‘keeping data private’. Another major challenge which makes e-professionalism difficult to constrain is that people’s personal lives spill into their professional examples include, class reunions, educational events, and networking flattens the contexts of SNSs [63,72]. Aase’s [19] narrative on e-professionalism as a big conversation with little or no control still holds true to some extent. Guraya et al.’s [72] recent research exalted this approach where participants articulated their inadequacies of self because of the unusual nature of the digital world. Balancing privacy, confidentiality, and ethical considerations.

Focusing on linguistic aspects, Lefebvre [41] reiterated the importance of protected health information and patient privacy in healthcare systems to prevent interactions being shared without regard for professional boundaries. Inversely, the reluctance to use SNSs for patient care can be misinterpreted as an inability of HCPs to communicate effectively with them [37,43,50,53,67]. Educating students and physicians to acquire a balance between professional yet distant, and depicting ‘to err is human’ in the digital world seems crucial [39]. The precarious nature of privacy and confidentiality in the online world gives rise to ethical issues from perpetual tracking of patient data to data manipulation [57]. Unfavourable or leaked data can compromise an organization’s reputation, resulting in the loss of the patients’ and the public’s trust in the HCPs [2,46,50]. To ensure that patients and their information is safe with HCPs, e-professionalism needs to be defined, described, and made explicit so that it can be practiced with clear boundaries in medical education [16,39,50,63].

### Logical principle: Does the concept under consideration maintain its distinct boundaries when theoretically integrated with other concepts?

The logical principle refers to the mechanism for integrating certain concepts into theory but remaining distinct from other relevant theoretical concepts [14,79,81]. Walker and Avant have argued that the testability of a theory is important for generating medical knowledge and for its evaluation if further research is required on the phenomenon being explored [9]. Furthermore, the soundness of a theory is critical when adopting it into practice shaping how problems are thought of in relation to health and medical care.

#### Theoretical foundations of e-professionalism

In this analysis, e-professionalism emerged as a relatively abstract concept encompassing several similar yet distinct boundaries with the concept of professionalism [82]. A complete understanding of e-professionalism can only be gained through a definition providing core elements of digital practices. Definitions solely stemming from the professionalism frame of reference limit the application of e-professionalism, ignoring context and negating new perspectives. In this area, theory testing can be accomplished either by qualitative or quantitative methods to determine the empirical adequacy [83].

#### Expanding boundaries and socio-cultural influences

At the concept boundary level, Ellaway [34] traced the roots of e-professionalism to traditional value-laden ­professionalism where altruism, honesty, integrity, accountability, excellence, duty, respect for others, and patient-centred service became misplaced in the web of SNSs. Although the importance of a behaviour-based professionalism framework may only be established through concrete evidence, the behaviours observed in the digital realm appear abstract and nebulous in nature. The work of Cruess and Steinert [84] and Chandratilake [85] produced a socio-cultural framework influenced by attitudes, beliefs, and in turn, behaviours. However, the omnipresence of an internet which is predicated upon the intermingling of virtual spaces has resulted in SNSs being neither fixed nor constant. A phenomenon should emerge comprising of human and nonhuman shared interactions. However, in actuality, an autonomous vacuum prevails. This amalgamation of private and public online lives creates a complex debate. The professionals and students participating in this debate have wildly differing generational perspectives.

#### Proficiency, responsibility, reputation, and transpersonal psychology

SNSs are often regarded as the best source of self-expression, but with skewed moral values and differing ethical standards prevailing among the current generation of HCPs, personal and professional boundaries have demolished hierarchies. Some may consider this to a be a cause for [38]. A medical student’s innocent online posts of images, videos, and text narrations of stories related to the workplace potentially jeopardizes the core principles of medical professionalism. This concept analysis revealed that students bought aspects of their social life into professional communities of practices. In this digital era, medical educators need to be attentive to students’ attitudes, behaviours, and online personas, thus necessitating the expansion of the professionalism paradigm [2,19,21,60,62,72]. Traditional definitions of professionalism concentrated on life as a professional while keeping one’s private life away from societal scrutiny. However, the e-professionalism perspective broadened the jurisdiction of traditional professionalism to both personal and professional lives highlighting the need of a new framework and a revision of the key attributes [71]. Keeping Clark’s extended mind theory [86] in focus, developing relationships with digital media has made SNSs an integral part of our problem solving and intelligence skills [2]. This new relationship has strengthened our cognitive abilities and critical skills signifying the need of a framework based on proficiency, reputation and responsibility, and transpersonal psychology [34,62]. The key attributes defined by Guraya et al. [62] help to build on these e-professionalism constructs.

Although digital natives [87] born and bought up in 90s found themselves in an environment saturated with digital technologies, they were found to be ill-equipped for digital literacy [18,62,74,75]. Gettig [27] identified two contrasting perspectives, students with a laxed approach to posting online content in a personal capacity while faculty wanted professionalism values to be maintained irrespective of personal and professional capacity. This disconnect between the beliefs of tech-savvy student posting unprofessional online content and the professional image being created by educators was disconcerting [45,67]. A clear need to procure the required professional digital behaviour was evident. Schroeder [48] urged the need for *professional digital behaviour discourse,* which remained elusive due to lack of clear frameworks and guidelines. Breaches of confidentiality, violations of boundaries, and unintended potential harm to patients demanded a cohesive characterization of e-professionalism [2,63,69]. Kleppinger and Cain [37] coined the term ‘*digital identity’* in digital communications to appease the frustration of educators due to the erosion of societal trust, individuals’ responsibility, and accountability [23]. Numerous medical associations have worked on producing good practice and best practice guides to help navigate the online world of SNSs [25]. However, Kang et al. [36] reported a lack of awareness of these guidelines by medical students. The absence of a prescriptive set of guidelines with an emphasis on key attributes of ‘e-professionalism’ and its compliance was evident [31].

Cain [20] described concerns about SNSs postings on three key aspects: reputation, privacy, and productivity, highlighting responsible digital behaviour. The digital context has become a confounding variable in the equation for effective online communication [71]. In addition, Spector [22] notion of Public, Permanent, and Powerful where online interactions are projected to variety of unknown audiences shows wide reaching repercussions [28]. In two studies by Jones et al. [40] and Jackson et al. [51] e-professionalism was considered an appropriate competency, but not through avoidance of social media by medical schools [60]. But rather a more proactive approach to inculcate reflective practice in the use of SNSs aimed to harbour metacognitive skills. Also, a shift from paternalism to autonomy to empower communities demands a heightened awareness of e-professionalism attributes, development of policies, remediation strategies, and curricular amendments to identify and track digital unprofessional behaviours [22,30].

## Discussion

This principle-based concept analysis was used to analyze the conceptual boundaries of e-professionalism in the field of medical education. This allowed the determination of the current state of knowledge surrounding e-professionalism. This literature review provides a clearer understanding of the conceptual basis of e-professionalism by delivering a clear basis for debating the importance of e-professionalism by HCPs.

Our concept analysis applied epistemological, pragmatic, linguistic, and logical principles to scrutinize the multifaceted dimensions of this evolving concept. The logical section elucidates the integration of e-professionalism within the broader context of professionalism while emphasizing distinct boundaries. The pragmatic analysis explored the practical utility of e-professionalism, examining its application in both academic and clinical settings. The linguistic component revealed the fledgling nature of e-professionalism, as well as the challenges in achieving a consensus around its meaning and consistent usage. Lastly, the logical lens exposed the gap in knowledge in understanding and defining e-professionalism, emphasizing the need for digital awareness among healthcare professionals. Together, these sections converge to offer a nuanced understanding of e-professionalism, paving the way for a robust and insightful discussion on the implications, challenges, and future directions in the digital landscape of healthcare professionalism.

**Figure 3.**
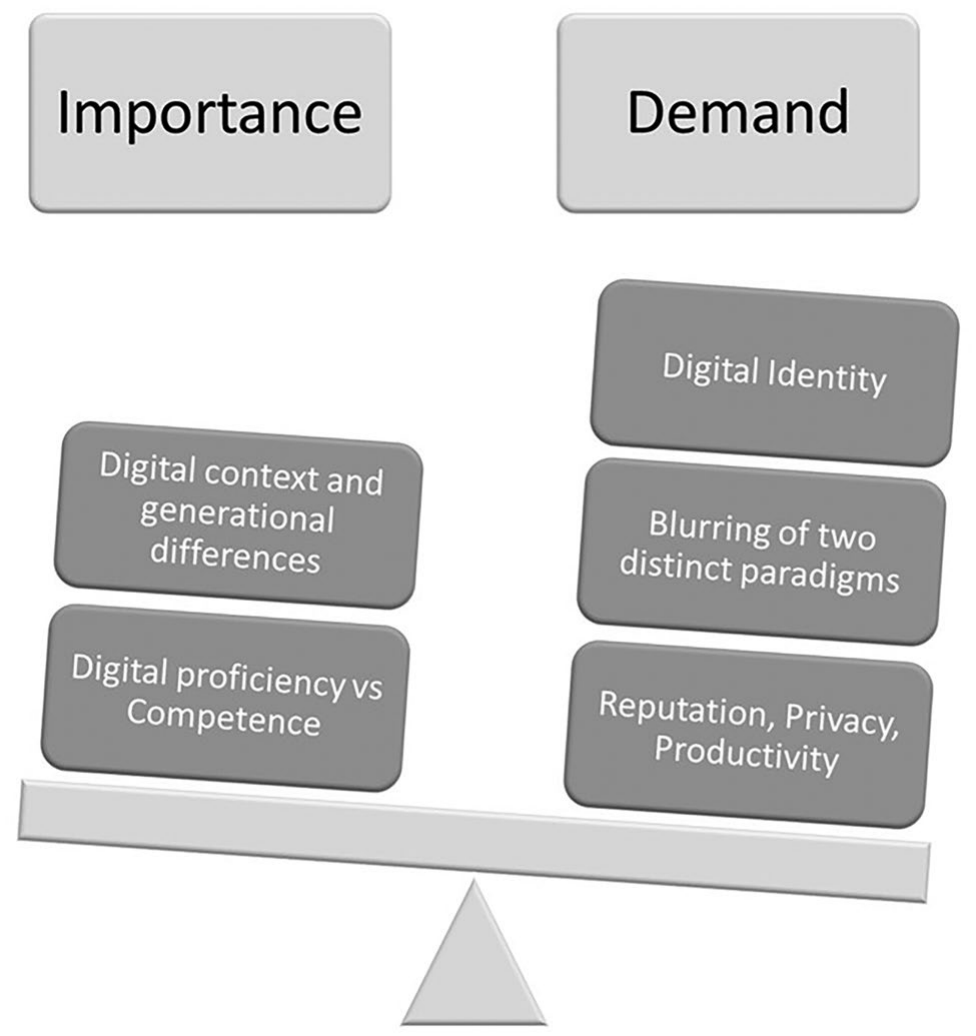
The importance and demand of e-professionalism.

This analysis provides a clear expose of e-professionalism giving rise to components to better explain and relate to the concept (Figure 3). To provide a working model of e-professionalism, core attributes arranged in the form of a framework encompassing the epistemological, ontological, and axiological aspects [62] would provide others with clear guidance.

Our concept analysis under the epistemological principle indicated a well-perceived concept of e-professionalism in all disciplines of the medical field. The concept of e-professionalism was found to be a clearly defined distinct entity and a recognizable area in medical education [17,62]. However, e-professionalism and its manifestations were being understood and interpreted inconsistently. Our work forms the foundation to produce a standardized unified application of e-professionalism for HCPs. This multi-construct frame of reference can then be used to compare, refine, and enumerate the desired attributes to be digitally professional [62]. From a pragmatic standpoint, the analysis of published literature revealed a mild maturity with an absence of clear methods to evaluate this concept. Therefore, further work is essential to clarify and understand the theoretical and operational utility of clinical medicine. Allowing us to further explore the use of sound methodological and philosophical paradigms while evaluating this concept. The linguistical lens revealed both consistencies and inconsistencies regarding the use, meaning, and depictions of e-professionalism. This is probably due to the rapidly changing technology and its unpredictable manifestations of digital professional behaviours. However, Guraya et al. [72] and Vukušić Rukavina et al. [74] have used this principle to guide the methodological context of their respective research. The logical principle highlights Cain and Romanelli [17] proposition of generational differences along with an element of philosophical nuances producing confusion of professional and personal boundaries in the digital space.

Despite having a better understanding of technological advancements than more senior HCPs, the younger generations have a different understanding of e-professionalism. They perceive that their private and personal lives do not fall under two jurisdictions. Conversely, older generations, although digital immigrants, quickly identified the singularity of identities necessary for the digital space. Hence, the digitally literate and digitally professional are two entirely different entities. There exists a clear understanding that technological advancements demanded different skills set acquisitions to understand boundary violations [42,44,62]. These include information ecosystem, self-disclosure, and the appropriate use of health services to maintain professional standards in an online environment [50]. Guraya et al. [63] advocated a proactive system-level approach to bridge the gap between policy makers and those using the technology for health care. A requisite for those using online technology should be further education with frequent reminders and appropriate role modelling to help evaluate and understand unprofessional digital behaviours. Educational opportunities should be identified which include online privacy [41], confidentiality [16,39], copyright, trade slander, libel laws, respecting boundaries [26].

## Limitations

Our literature synthesis on e-professionalism in medical education had potential limitations that warrant consideration. First, the sampling bias in the reviewed literature might not have captured all perspectives. Second, the discipline-specific nuances may not have been captured comprehensively, a potential limitation in the generalizability of our results. Third, the chosen conceptual framework of Penrod and Hupcey may have inherent limitations. Fourth, the rapid evolution of technology may render some findings outdated. Finally, the temporal constraints and subjectivity of the research team could influence our interpretation. Acknowledging these limitations was important for guiding future research endeavors in this dynamic field.

## Conclusion

This concept analysis reports the current state of knowledge around e-professionalism and provides insights on how to advance this field. In doing so, we describe a philosophical evaluation of the existing research providing a valuable set of findings and a roadmap for future research.

The summative findings revealed that e-professionalism is a moderately mature concept, with a unanimously agreed-upon definition. Lapses of e-professionalism judgement were apparent in the existing body of research, highlighting the destructive nature of digital media on the professional behaviours and attitudes. Infringements of e-professionalism behaviour arose from Z-generation’s need for connectivity and communication of medical information. While it is understood differently across generations and cultures, this concept analysis identified an explicit need for delineation of essential attributes of e-professionalism to aid future HCPs. Unfortunately, validated measurements tools for evaluation and remediation of e-professionalism lapses are still in their infancy.

## Supplementary Material

Supplemental Material

## Data Availability

The raw dataset and other materials are available on request. The corresponding author will provide additional data if requested.
